# To Provide a Double Feeder in Growing Pigs Housed under High Environmental Temperatures Reduces Social Interactions but Does Not Improve Weight Gains

**DOI:** 10.3390/ani10122248

**Published:** 2020-11-30

**Authors:** Tâmara Duarte Borges, Mariana Huerta-Jimenez, Nicolau Casal, Joel Gonzalez, Nuria Panella-Riera, Antoni Dalmau

**Affiliations:** 1 School of Life Science, Graduate Program in Animal Science (PPGCA), Pontíficia Universidade Católica do Paraná—PUCPR, Rua Imaculada Conceição, 1155, Prado Velho, Curitiba 80215-901, PR, Brazil; tamaratdb@hotmail.com; 2 Faculty of Animal Science and Ecology, Autonomous University of Chihuahua, Perif. Francisco R. Almada km. 1, Chihuahua 31453, Mexico; mhuertaj@uach.mx; 3 IRTA Veïnat de Sies S/N, 17121 Monells, Spain; nicolaucasal@gmail.com (N.C.); joel.gonzalez@irta.cat (J.G.); nuria.panella@irta.cat (N.P.-R.)

**Keywords:** body weight, carcass quality, growing pigs, meat quality, social behavior, thermal stress

## Abstract

**Simple Summary:**

Competition for food can increase if pigs concentrate feeding times in the cool hours of the day during the warmest seasons. The present study addresses whether providing the animals with a double feeder would benefit the performance of pigs when subjected to high environmental temperatures. The results showed that high environmental temperatures reduced the final body weight of pigs and increased the percentage of fat in the carcass of the animals. When provided with two feeders instead of one, the animals reduced the number of social interactions, so less competition for food was achieved. However, an unexpected result was a decrease in the body weight of the animals with two feeders when compared to the animals with one, so in terms of performance, the strategy failed in providing the expected results.

**Abstract:**

Heat stress and competition for food are two major challenges in pigs reared in intensive conditions. The aim of the present work was to study the effect of providing a double feeder for pigs reared under two different environmental temperatures. In addition, two types of flooring, of 100% slat and 30% slat 70% concrete, were also considered. A total of 256 pigs in the growing-finishing period (from 27 kg to 110 kg) were housed using two environmental temperatures: control (from 18 °C to 25 °C) and heat stress (above 30 °C six hours a day). They were housed in 32 pens of 8 pigs each, distributed into 4 rooms (16 with one feeder and 16 with two). Pigs subjected to temperatures above 30 °C up to six hours had lower body weight gains than pigs subjected to a maximum temperature of 25 °C, confirming that thermal stress negatively affects performance in pigs. In addition, heat stress affected the final product by decreasing the lean percentage of carcasses by 2.6%. A double feeder reduced the presence of negative social behavior, especially in the feeding area, but body weight was lower than when one single feeder was used. A 30% slat 70% concrete floor showed better results in the pig stress indicators and body weights than 100% slat. It is concluded that providing a double feeder in the pens, although reducing the presence of negative social interactions, negatively affected body weight, in comparison to pigs fed with just one feeder.

## 1. Introduction

According to the Intergovernmental Panel on Climate Change (IPCC, 2007) [1], warming of the climatic system is unequivocal, and it estimates a confidence level of >90% that there will be more frequent warm spells and heat waves. The production of pork takes place mainly in areas where the temperature of the environment is periodically over the thermoneutral range of pigs, and there is a severe fluctuation in day temperatures to which the animals have to adapt [2]. As a result, the risk of heat stress is more frequent and severe in pork production than ever before [3]. Heat stress produces important industry losses, reaching over $300 million annually in the European Union [4]. Pigs are particularly sensitive animals to high temperatures because of the low ability to lose latent heat. For instance, pigs do not sweat. In addition, the most used breeds for production have been subjected to intensive genetic selection to obtain greater muscular ability [5], increasing the capacity of the animals for producing heat. In order to maintain homeostasis, the body temperature of pigs should stay within a range of 38 °C to 39 °C.

One of the most important abiotic stress agents in animal husbandry and feeding is heat stress, which, according to Jócsák et al., 2020 [3], occurs when the ambient temperature rises above 25 °C. As a consequence, cardiac function accelerates and peripheral circulation increases [6], and voluntary feed intake decreases [7,8]. Heat stress had been reported to induce immunological effects in growing-finishing pigs, where blood cells may enhance cell-mediated immune responses while suppressing humoral responses or vice versa, thus disrupting the balance between these components of the immune system, principally the neutrophil/lymphocyte ratio (NLR) [9]. This ratio combines two independent markers of inflammation that can be used to assess an animal’s difficulty in coping with the environment [10]. Similarly, hair cortisol evaluates chronic stress, and can be used to assess the difficulty of animals in coping with different type of challenges [11].

Pigs use few strategies to dissipate heat, with most being behavioral [12]. One of them is to look for fresh bathing areas to increase evaporative heat loss [7,13]. However, in the pens of intensive production systems, usually the only wet area is where the animals defecate and urinate, and the available surface for that depends of the floor type. In fact, in Dalmau et al. (2019) [14], it is described how a floor of 30% slat and 70% concrete allows the pigs to cope better with high environmental temperatures than 100% slat. This is explained because a measure of 70% concrete allows the animals to lie easily on feces and, consequently, become wet [14].

Another strategy that pigs can use is to change their feeding rhythms, which means to concentrate feeding periods in the cool hours of the day [15]. However, in the cases where access to food is restricted, this may produce greater competition for food, forcing the subordinate animals to eat in the hottest hours. This can produce a reduction in food intake and therefore a loss in performance of these animals [16]. Heat stress can also be associated with meat quality in the different quantity and distribution of fat [17]. Animals in a heat-stress environment can consume about 12.3% less food than can those in thermal comfort, with lower retention of protein and fat in the carcass [18,19], which could negatively interfere with meat texture and even with its water-holding capacity [20].

The aim of the present study was to compare behavior, performance, and meat quality in pigs subjected to heat conditions (above 30 °C six hours a day) in relation to lower temperatures (from 18 °C to 25 °C) when maintained on different floor types and different ratios of animals per feeder. In fact, fifty percent of the animals were provided with just one feeder (8:1) and the other half with two feeders (4:1). The hypothesis of the study was that by providing a double feeder, the competition for feeding would be reduced, so animals could better decide at which time of the day they would eat. In consequence, the warmest periods of the day would be avoided, increasing their food consumption, thus obtaining better results on body weight gains.

## 2. Materials and Methods

### 2.1. Animals and Experiment Design

The study was conducted in compliance with the European guidelines for use of animals in research, and the protocol was approved by the Ethical Animal Committee (IACUC) of the Institut de Recerca i Tecnologia Institute of Agrifood Research and Technology (IRTA, Barcelona, Spain; 2014/8348). A total of 256 pigs in the growing-finishing period were used in this study. All pigs, a three-breed cross of Large White × Landrace and Pietran, were reared up to 28 kg ± 0.4 kg in a commercial farm and then transported to the IRTA facilities in Monells (Girona, Spain), where the study was performed. Fifty percent of the animals were females and the other fifty percent males upon arrival at the experimental facilities. Males where vaccinated against the Gonadotrophin releasing factor (GnRF) at weeks 6 and 9 after arrival at the experimental farm by the injection of 2 mL of Improvac^®^ subcutaneous (Zoetis, Spain).

**Figure 1:**
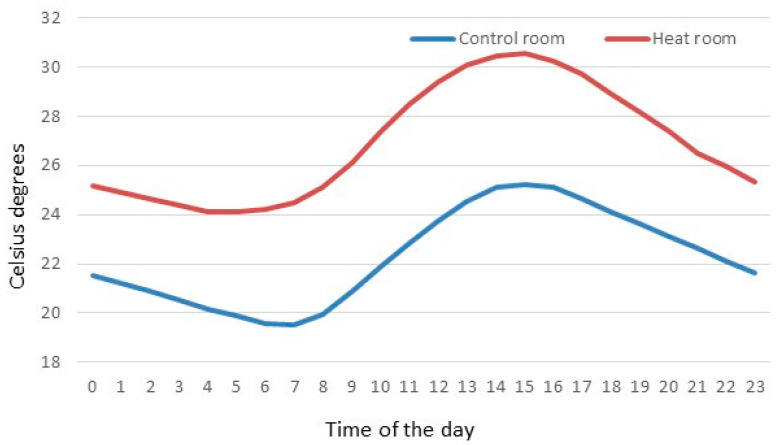
Average temperatures from 00:00 h to 23:00 h for control and heat rooms along the study.

The study was carried out during summer-autumn 2014 using a factorial experimental model of 2 × 2 × 2, considering: (a) two environmental temperatures: control (from 18 °C to 25 °C) and heat (above 30 °C up to six hours a day; Figure 1); (b) two types of flooring: with a totally slatted floor (100% slat) and a partially slatted concrete floor (30% slat 70% concrete); and (c) two conditions of feeder availability: one feeder per pen (one for eight animals; 55 × 37 cm) or two feeders per pen (one for four animals; (55 × 37 cm) × 2) and in both cases with ad libitum access to feed and water.

A total of 32 pens distributed in four rooms (A, B, C, and D) were used (four pens per treatment × eight treatments), with a total space of 6.75 m^2^. A feeder occupied 0.2 m^2^ (two feeders, 0.4 m^2^), so space allowance per pig was 0.82 m^2^ with one feeder and 0.79 m^2^ with two feeders.

**Figure 2:**
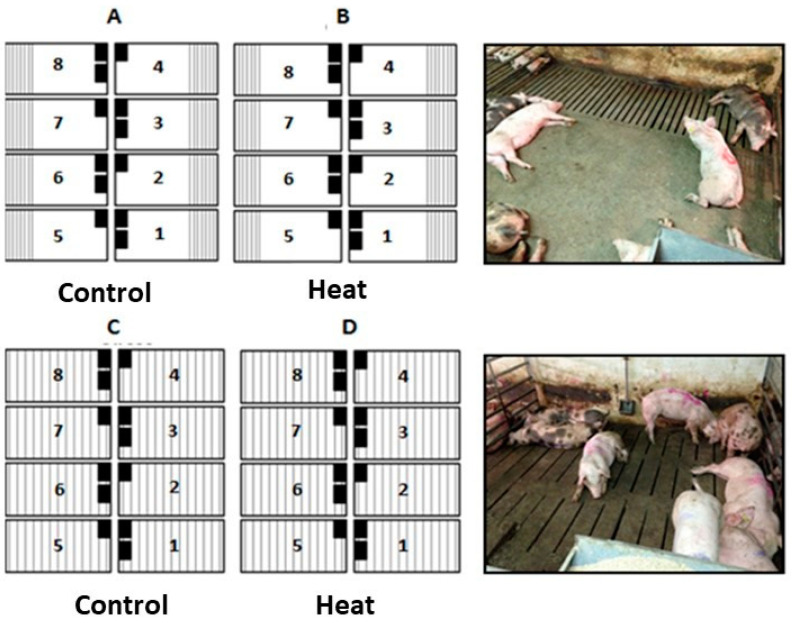
Scheme of the experimental design, considering the combination of two environmental temperatures (control and heat), type of floor (30% or 100% slat), and number of feeders (one or two). Room A had a 30% slatted floor, combined with a heat treatment; Room B, a 30% slatted floor with control temperature; Room C, 100% slatted floor with heat, and Room D, 100% slatted with control temperatures. All pens had eight animals inside, four females and four (vaccinated with Improvac^®^), and vaccinated with Improvac^®^ and the same number of pens with one or two feeders inside.

Rooms A and B had 30% slatted floors and rooms C and D 100% slatted floors. In addition, Rooms A and C were subjected to control temperatures while Rooms B and D to heat (Figure 2). Pens were numbered in all cases from 1 to 8 and all of them contained eight pigs (individually marked as well with a number from 1 to 8). Each pen housed four males (numbered from 1 to 4) and four females (numbered from 5 to 8). Animals were also distributed in a balanced way according to their initial weight. In fact, the four heaviest males were allocated to Pen 1 in Rooms A, B, C, and D. Then, the next four males in terms of body weight were distributed again in Pen 1 of Rooms A, B, C, and D. In consequence, body weight variability between animals in the same pen and among treatments at the beginning of the study was minimized. In contrast, the mean body weights of Pens 1, 2, 3, and 4 of each room was higher than those found in Pens 5, 6, 7, and 8. So, at the end of the study, animals were sent to the slaughterhouse in two batches (one containing pigs from Pens 1 to 4 and the other containing pigs from Pens 5 to 8 of the four rooms).

### 2.2. Body Weight

Pigs were weighed before beginning the experiment at the farm of origin (W1), 4 weeks after their arrival (W2), 4 weeks later (W3) and, finally, before being transported to the slaughterhouse (W4). Animals were slaughtered at 12 weeks after arrival at the facilities.

### 2.3. Blood samples

Blood samples from all animals were collected at the beginning (pigs at 28 kg, on average) and at the end of the experimental period (pigs at 100 kg, on average). Samples were collected from the jugular vein by trained veterinary technicians, using an EDTA tube, and were kept refrigerated at 4 °C until arrival at the laboratory 2 h later for immediate processing. Haematological parameters: packed cell volume (PCV), haemoglobin (Hb), red blood cells (RBC), and white blood cells (WBC), with relative counts (neutrophils, lymphocytes, eosinophils, basophils and monocytes) were analyzed immediately using an automatic counter. The neutrophils/lymphocytes ratio (NLR) was then considered as an indicator of chronic stress [9,10].

### 2.4. Hair Samples

At the arrival to the experimental facilities, weighing 28 kg, an area of around 10 cm × 10 cm located in the dorsal pig’s rump (10 cm from the tail) was shaved. Later, at the end of the study, when pigs weighted 100 kg, animals were sampled in the same area, taking advantage of the restraint provided by the cage of the weighing-scale. Hair was collected by shaving close to the skin with clippers, trying not to remove the root of the hair, and avoiding inclusion of the hair follicle in the sample. Once sampled, hair was stored at room temperature (22 °C ± 2 °C) inside hermetically sealed bags until analysis. Only 50% of the animals were sampled, although balanced by treatments (one or two feeders, control vs. heat room, and 30% vs. 100% slat).

Cortisol extraction was performed following the method of Davenport et al. (2006) [21] with minor modifications. First, approximately 150 mg of hair were washed twice in 3 mL of 99.5% isopropanol for 30 s to eliminate contaminants that could interfere with the determination of cortisol (in case of smaller samples, the volume of isopropanol was reduced to maintain the same proportion). The hair was then allowed to dry overnight in the airflow hood. The next day, samples were finely minced using surgical scissors until hair segments were 0.3 cm maximum length. For cortisol extraction, 1 mL of 99.5% methanol was added to approximately 50 mg of powdered hair, and it was incubated at 37 °C for 17 h with slow rotation. Then, the sample was spun in a microcentrifuge for 30 s at 5000 rpm. At the end of extraction, the eppendorfs were centrifuged, and 0.6 mL of the supernatants were finally dried using a vacuum centrifuge, and stored at 20 °C. The dry extract was reconstituted in phosphate buffer solution from the assay kit.

### 2.5. Pig Behavior

Behavior was recorded in two ways. Detailed records were made by direct observation on five weekdays during the animals’ growing-finishing period (10 weeks in total), and video recording was used to monitor feeding-activity patterns during a 72 h continuous period.

**Figure 3:**
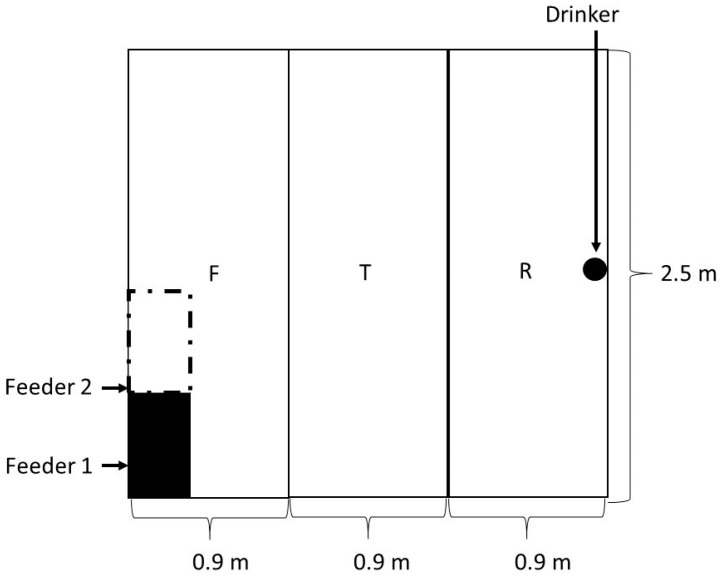
Areas into which the pen was divided to assess the social interactions among pigs.

For direct behavioral observations, each pen group of eight pigs was observed daily by means of a 5-min focal sampling by two observers trained previously according to the Welfare Quality methodology [22]. In total, 1536 5-min observations for the 32 pens (48 per pen) and moment of the day: morning (M—from 7:30 h to 10:00 h), heating period (H—from 10:01 h to 16:00 h), and afternoon (A—from 16:01 h to 18:30 h), with 512 observations each, were performed. The observations focused on positive and negative social interactions according to the definition of Welfare Quality, where positive interaction is defined as sniffing, nosing, licking, and moving gently away from the animal without aggressive or flight reaction from this individual, and negative interaction is defined as aggressive behavior, including biting or aggressive social behavior with a response from the disturbed animal [23]. The assessments were carried out from the corridors, trying to avoid any disturbance to the animals. In addition to the social interaction by itself, the area inside the pen where these interactions took place was registered as well, with three possibilities considered: R zone, which was the area used commonly for resting and closest to the drinker; T zone, which was the transitional area allocated in the middle of the pen; and F zone, which was the area closest to the feeders (Figure 3). In case of doubt, the area where an interaction was registered was decided according to the position of the actor of this activity and not according to the receiver. To be considered in an area, more than half of actor pig’s body had to be located inside of this location.

Pig feeding behavior from 16 pens in total (8 with one feeder and 8 with two feeders, 8 in control room and 8 in heat room, and 8 in 30% slat and 8 in 100% slat) was recorded on videotape, over 3 consecutive days (4 times per day, at 06:00 h; 12:00 h; 18:00 h and 24:00 h) 2 weeks before slaughtering the animals and was analyzed. The cameras were allocated between Pens 1 and 2 and 5 and 6 in each of the 4 rooms, providing a good view of the feeding area (F zone, Figure 3). During dark hours, low-intensity red lights provided sufficient light for video recording. Recordings were later reviewed in real time to ascertain the feeding behavior of the pigs in each treatment. In this case, the total duration of a meal was considered.

### 2.6. Carcass and Meat Quality

Twelve weeks after the arrival to the facilities in Monells, pigs were transported 140 km to a commercial slaughterhouse. They spent around 4 h in the lairage pens, allocated in groups of 32 animals. Pigs within the same room were mixed, but not animals from different rooms. The pigs were stunned by application of CO_2_ at high concentrations (90% for 2 min and a half) and slaughtered according to common commercial practices.

For carcass and meat-quality analysis, only pigs from Pens 5 to 8, containing a total of 128 animals, were used. Carcasses were weighed warm after their splitting, at 20 min post-mortem by using an online scale to calculate carcass yield. Lean meat percentage (LMP) was measured at the slaughterhouse using VCS2000 carcass grading equipment. Meat-quality measurements were carried out in the left-half carcass of the animals. The pH was measured in the longissimus thoracis (LT) muscle at the last rib level at 1 h (pH_1_) and 24 h (pH_u_) post-mortem, using a portable pH meter (Crison, Hach Lange Spain S.L.U.; Spain) equipped with a Xerolyt penetration probe. Electrical conductivity was measured in the LT using a conductimeter (PQM Future, Classpro GmbH; Germany) at the last rib level at 1 h (EC_1_) and 24 h (EC_u_) post-mortem. Meat color was determined at 24 h post-mortem on loin samples from each animal slaughtered. A colorimeter CR-200 (Konica Minolta Inc.; Tokyo, Japan) was used to obtain the lightness (L*) of meat, defined by the Commission International de I’Eclairage (CIE; 1976) [24].

Drip loss was determined from the LT muscle according to the methodology described by Rasmussen and Andersson (1996) [25], placing duplicate meat samples from each animal in sealed plastic tubes at 4 °C, which is directly related to the exudation of meat. Six cores were obtained from each loin sample and were used for shear-force measurement. Shear force was determined using a texturometer (Stable Micro Systems Ltd.; Godalming, Vienna Court, Lammas Rd, Godalming GU7 1YL, UK) and the Warner-Bratzler test. Intramuscular fat (IMF) of the LT from the last rib level was determined by near infrared spectroscopy (Foodscan, FOSS; Denmark).

### 2.7. Statistical Analysis

Statistical analyses were performed by means of the statistical analysis system (SAS) (SAS 9.1; software SAS Institute Inc.; Cary, NC). Data from meat and carcass quality, such as carcass yield, lean meat percentage, pH_1_, pH_u_, EC_1_, EC_2_, IMF, L*, drip loss, shear force and IMF, packed cell volume, hemoglobin, total red blood cells, number of neutrophils and number of lymphocytes were analyzed using the PROC MIXED procedure with pen as random effect. The models accounted for the effects of environmental temperature (heat rooms vs. control), floor type (30% slat vs. 100% slat), number of feeders (1 vs. 2), gender (males vs. females), and possible interactions. The hot-carcass weight was included as a covariate in the models where needed. The residual maximum likelihood was used as a method of estimation. The least square means of fixed effects (LSMEANS), adjusted to Tukey’s honestly significant difference (HSD), was used to carry out multiple comparisons. Non-parametric variables, such as body weight, total white blood cells, number of eosinophils, basophils, monocytes, NLR, cortisol, and social interactions were analyzed with PROC GENMOD with Poisson or negative binomial distributions according to Cameron and Trivedi [26], except in the case of the positive/negative social interactions ratio, where a binomial distribution was used. The models accounted for the same effects mentioned previously for PROC MIXED, and LSMEANS was used to carry out multiple comparisons. Pens with fewer than eight animals, which occurred in just one case due to the death of one animal, were not considered for performance analysis. Significance was fixed at *p* < 0.05 in all cases.

## 3. Results

### 3.1. Body Weight

**Figure 4:**
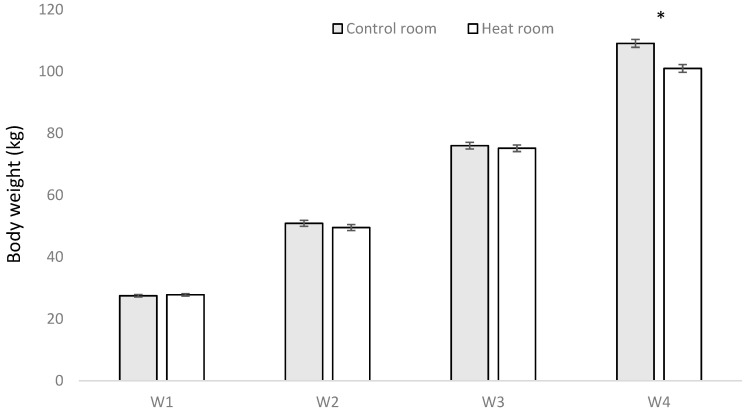
Mean ± SE of pig body weight per period (W1, W2, W3, and W4) comparing heat and control rooms. * means significant differences at *p* < 0.05.

**Figure 5:**
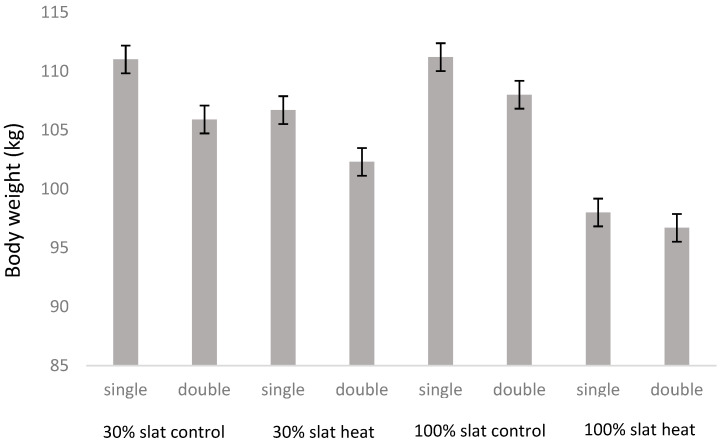
Mean ± SE of pig body weight at the end of the study depending of the environmental temperature (control vs. heat), type of floor (30% slat 70% concrete vs. 100% slat), and number of feeders per pen (one: single; two: double).

No significant effect was found for treatment (heat and control conditions), type of floor (30% or 100% slat), or interactions between both factors for body weight assessed at the farm of origin (W1), four weeks after arriving at the facilities of the study (W2), and one month prior to the end of the study (W3, Figure 4). However, the final weight before being transported to the slaughterhouse (W4) was higher (*p* < 0.0001) for animals in the control room, in comparison to animals in heat rooms (Figure 4), and in animals housed in 30% slat (106.4 kg ± 1.21 kg body weight) in comparison to animals housed in 100% slat (103.0 kg ± 1.15 kg body weight; *p* = 0.0387). In fact, the average daily gain was only different between heat and control conditions in the period from W3 to W4 (*p* = 0.0245), being 1.01 kg per day in control room and 0.96 kg per day in heat room, a similar difference (*p* < 0.0001) to those found between 30% slat (1.01 kg per day) and 100% slat (0.95 kg per day; Table 1). From period W1 to W2 this value was of 0.77 kg per day and in the period W2 to W3 0.82 kg per day. In this final weight, as occurred in W1, W2, and W3, no effect was found for the double interactions of floor type with heat, floor type with number of feeders, or heat with number of feeders (Table 2). However, a triple interaction of heat, type of floor, and number of feeders was found (*p* < 0.0001; Table 2). In fact, in heat rooms with 100% slat there were no differences between pens with one or two feeders (Figure 5).

In relation to the number of feeders, no differences were found between having access to one or two feeders at the beginning of the study (W1). Nevertheless, already in W2, an effect was found of number of feeders (*p* = 0.0236), with animals in one-feeder pens being heavier (51.4 kg ± 0.31 kg) than in two-feeder pens (49.0 kg ± 0.28 kg). This effect was also seen in W3 (*p* = 0.0135), the body weights being 77.3 kg ± 0.57 kg and 73.9 kg ± 0.65 kg for pens with one and two feeders, respectively, and W4 (*p* = 0.0234), the body weights being 106.5 kg ± 1.01 kg and 101.8 kg ± 0.83 kg for pens with one and two feeders, respectively. Accordingly, the average daily gain was higher when the animals had one feeder than two feeders from W1 to W2 (0.80 and 0.75 kg per day, respectively; *p* = 0.0377), from W2 to W3 (0.84 and 0.80 kg per day, respectively; *p* = 0.0135), and from W3 to W4 (1.00 and 0.96 kg per day, respectively; *p* = 0.0443; Table 1).

### 3.2. Hematological Parameters and Hair Cortisol

Packed cell volume, hemoglobin, red blood cells, and white blood cells, including relative counts of neutrophils, lymphocytes, eosinophils, basophils, and monocytes at the end of the study are shown in Table 3. No effect of number of feeders was found for any variable, but a type of floor effect (*p* < 0.01) was found for global white blood cells, neutrophils, and lymphocytes (Table 2). In addition, a temperature effect (*p* < 0.001) was found for eosinophils and monocytes (Table 3).

**Figure 6:**
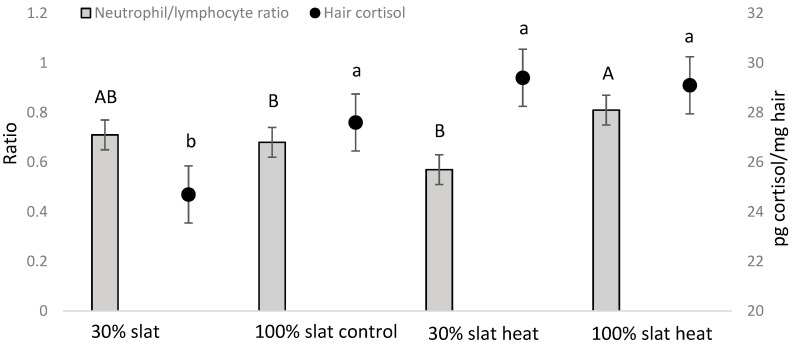
Mean ± SE of neutrophil/lymphocyte ratio (in bars) and hair cortisol (points) in pigs at the end of the study depending of the environmental temperature (control vs. heat) and type of floor (30% slat 70% concrete vs. 100% slat). Different letters mean significant differences at *p* < 0.05.

For the first sampling of blood taken before the beginning of the study, no effect of neutrophil/lymphocyte ratio (NLR) was found between the different factors studied (heat, type of floor, or number of feeders). However, in the second blood sample taken at the end of the study, although no effect of number of feeders was observed (NLR being 0.71 ± 0.07 and 0.72 ± 0.07 for one and two feeders, respectively) an interaction between heat and type of floor was found (*p* = 0.007; Table 2). The animals from the heat room with a 100% slatted floor had a higher ratio than the animals from the control room with a 100% slatted floor and the animals from the heat room with a 30% slatted floor (Figure 5). Hair cortisol analysis did not show differences between number of feeders (being 24.5 ± 1.01 and 26.4 ± 1.06 pg of cortisol/mg hair for one and two feeders, respectively) and only showed an effect in rooms with 30% slatted floors, the cortisol values being lower (*p* = 0.0492) in the control room than in the heat room (Figure 6; Table 3).

### 3.3. Behavioral Observations

**Figure 7:**
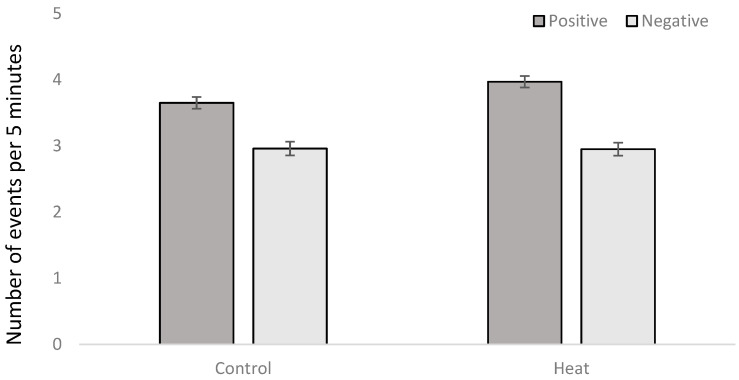
Mean ± SE of number of positive social interactions and negative social interactions per observational period of five minutes depending of the environmental temperature (control vs. heat).

A total of 9965 interactions (1.3 per minute) were found, 5619 being classified as positive social interactions (56% of the total interactions) and 4346 as negative social interactions (44% of the total interactions). An effect of number of feeders (*p* = 0.0008) and type of floor (*p* = 0.0005) but not interactions was found for negative social behavior (Table 2). Pens with just one feeder had more events of negative social behavior (3.11 ± 0.108 events per observational period of five minutes) than pens with two feeders (2.80 ± 0.095 events per observational period). In addition, pens with a 30% slat also had more events of negative social behavior (3.09 ± 0.105 events per observational period) than pens with 100% slat (2.82 ± 0.099 events per observational period). An effect of heating or not heating the room (*p* = 0.0013) was found for positive social behavior, heat rooms having more events (3.97 ± 0.089 events of positive social behavior per observational period) than control rooms (3.65 ± 0.086 events per observational period; Figure 7).

**Figure 8:**
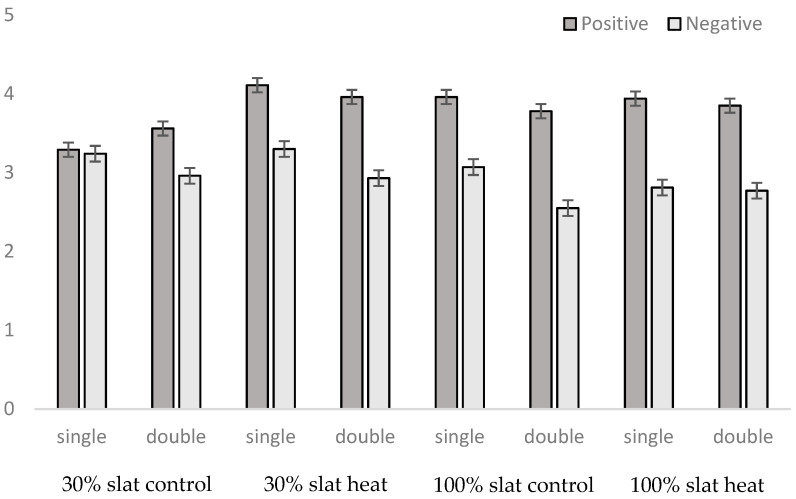
Mean ± SE of number of positive social interactions and negative social interactions per observational period of five minutes depending of the environmental temperature (control vs. heat), type of floor (30% slat 70% concrete vs. 100% slat), and number of feeders (one: single; two: double).

In addition, an interaction between heat and type of floor (*p* = 0.0030; Table 1) was found for positive social behavior, the heat room with a 30% of slat showing a lower presence of positive social behavior than any other room (Figure 8).

**Figure 9:**
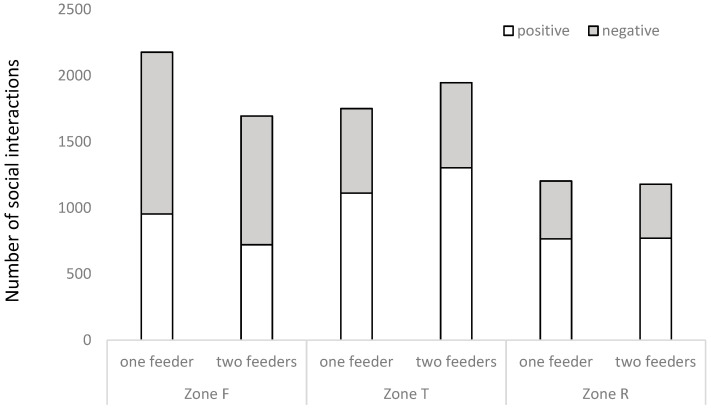
Presence of positive and negative social interactions in relation to the zone of the pen (where Zone F is the feeding area, Zone T is the intermediate area, and Zone R is the resting area) when pens had one feeder or two feeders.

The global positive social interaction/negative social interaction ratio (being 1.29 globally) was affected by place inside the pen (*p* < 0.0001), the moment of the day (*p* < 0.0001), heat (*p* = 0.0065), floor type (*p* < 0.0001), and feeder number (*p* = 0.0018). In Areas R and T, 65% of the interactions were positive, while in Area F, only 43% of the interactions were registered as positive (Figure 9). In the morning, 59% of the interactions were positive, while at midday and in the afternoon it was just 54%. In the heat rooms, 58% of the interactions were positive, while in the control rooms it was just 55%. With a 30% slatted room, 55% of the interactions were positive, while with 100% slatted, it was 58%. Finally, with one feeder, 55% of the interactions were positive, while with two feeders this reached 58%. In terms of feeders, animals allocated with two feeders had fewer (*p* < 0.0001) interactions in the feeding area than animals with just one (Figure 9).

According to the video recordings, animals in a 30% slatted room had shorter meals (*p* = 0.0170) than animals in a 100% slatted one (87.2 s ± 3.96 s and 108.2 s ± 4.70 s, respectively). In addition, with a double feeder the animals had shorter meals (*p* = 0.0234) than those with one feeder (91.9 s ± 4.90 s and 106.6 s + 5.11 s, respectively).

### 3.4. Carcass and Meat Quality

Most of the parameters assessed in the present study in terms of meat and carcass quality, such as carcass yield, intramuscular fat, pH at 45 min after slaughter (pH_1_), electrical conductivity at 45 min after slaughter (EC_1_) and at 24 h (Ec_u_), color, drip loss, and shear force were not affected by the environmental temperature treatments applied, floor type, or number of feeders in the pens (Table 2; Table 4). However, the percentage of lean meat on the carcass and pH at 24 h after slaughter (pH_u_) were significantly higher (*p* = 0.043 and *p* = 0.009, respectively) in control rooms, as compared to heat rooms (Table 4). An interaction between floor type and environment temperature was also found (*p* < 0.001) for lean percentage. In fact, in the control room with 100% slat, leaner animals were obtained, as compared to animals in the control room with 30% slat (54.7% ± 3.83% and 49.7% ± 1.71% for 100% and 30% slatted, respectively), while the contrary was found under heat conditions, with animals reared with a 100% slatted room having lower lean values than those reared in 30% slatted (48.5% ± 2.87% and 50.7% ± 0.87% for 100% and 30% slatted, respectively).

## 4. Discussion

Pigs are particularly sensitive animals to high temperatures because of the low ability to lose latent heat. Although Jócsak et al. (2020) [3] defined when pigs were subjected to temperatures above 25 °C as thermal stress, Berton et al. (2015) [28] considered optimal temperatures for growing and finishing pigs to be 16 °C and 20 °C, respectively. Therefore, under the conditions of the present study, where maximum temperatures of 25 °C were achieved in the control group, this should not be considered absolutely free of some thermal stress (Figure 1). In the heating treatment, animals were subjected to temperatures above 30 °C up to six hours a day, with a maximum temperature registered of 32.03 °C, and the rest of the day temperatures were maintained around 25 °C (Figure 1). However, even under these two treatment conditions, we found an effect of heat stress in the neutrophil/lymphocyte ratio and hair cortisol, confirming that for animals the heat rooms were, in general, worse than the control ones. In this case, hair cortisol is of special interest, because it allows to ascertain the chronic stress that an animal suffers during the entire period that the hair is growing [21]. In fact, every day a small representation of the cortisol concentrations of the animal is retained in the hair, so the results show the accumulative effect of stress on the animals. However, under conditions of chronic heat stress, it could be expected that cortisol would decrease, since higher levels of cortisol are associated with higher metabolic heat production, which is counterproductive in heat stress conditions. Therefore, it could be the chronic psychological stress that caused this increase of cortisol. One of the factors of this psychological stress is social competition for resources. However, although positive social behavior (defined as sniffing, nosing, licking, and moving gently away from an animal without aggressive or flight reaction from this individual [23]), was higher in heat rooms than control rooms, no differences were found between treatments in the presence of negative social behavior (defined as aggressive behavior, including biting or aggressive social behavior with a response from the disturbed animal, [23]), so this specific cause for psychological stress should be discarded. In any case, in this study and according to the physiological indicators, the best scenario for pigs in the long term was 30% slat 70% concrete under control temperatures.

At the end of the study, finishing pigs housed in heat rooms weighed 7.9 kg less than pigs housed in control rooms. In fact, in their strategies to cope with high environmental temperatures, pigs reduce heat production [29,30], with reduction in voluntary feed intake [30,31] and, in consequence, body weight. The results of Collin et al. (2001) [32] demonstrate a 30% decrease of voluntary feed intake in finishing pigs (>50 kg) at 33 °C, associated with shorter daily ingestion times (−28%) and consumption times (−34%). With growing pigs (25 kg to 50 kg), the negative effects of heat stress are not as great as with heavier finishing pigs [33]. This agrees with the results obtained in the present study, as the only significant differences between pigs of both temperature treatments was found in the last weighing (Figure 4).

In addition, a floor of 70% concrete and 30% slat resulted in a higher final body weight than a 100% slatted floor. This is discussed in Dalmau et al. (2019) [14], where data are presented regarding how a higher presence of concrete allows the animals to become dirtier, and this could influence the capacity of pigs to cope better with high temperatures by increasing heat losses across evaporation on a wet skin. Additionally, according to Pedersen and Ravn (2008) [34], a solid floor provides more stability to pigs and it is more comfortable to rest upon [35]. For instance, an increased risk of abnormal gait in animals housed on slatted floors, compared with solid concrete floors, has been described [36]. Accordingly, the worst combination for animals resulted to be the heat room with 100% slat, having the worst values of body weight (Figure 5) and stress indicators (Figure 6). On the other hand, it was the only case where no differences were found between one and two feeders.

An unexpected result was the fact of obtaining worse performance, already at the second weighing of the study (four weeks after beginning), when two feeders were used per pen in comparison to use just one. Traditionally, the feeder space requirements for pigs are 1 space per 10 animals [37,38]. Baxter (1991) [39] suggested that the minimum width of a feeding space should be the shoulder width of the pig, plus 10% to accommodate pig variability and movement. This is around 30 cm for a pig of 100 kg. Gonyou and Lou (1998) [40] suggest that feeder depths for growing-finishing pigs should be 20 cm to 30 cm. In the present study, feeders were 55 cm × 24 cm, so just with one feeder per eight animals, the conditions were optimal for pigs, and in pens with two feeders they reached 110 cm × 24 cm of feeder available, so the situation was even better. It is known that in all species, environmental conditions change feeding patterns [15]. For instance, Cross et al. (2020) [41] described how, during elevated temperatures, pigs increased feeding events during the early (03:00 h–05:59 h) and late (18:00 h–20:59 h) periods of the day. The hypothesis of the present study is based on the fact that high temperatures concentrate the feeding times in the cool hours of the day, so providing two feeders instead of one, even in small groups of eight pigs where the animal per feeder ratio is already optimal, would reduce the competition for this resource and would benefit the pigs and their capacity to cope with heat.

In the present study, the double feeder reduced negative social interactions (Figure 8) in comparison to pens with just one feeder, and this effect was caused by the reduction of social interactions in the feeding area, but not in other areas of the pen (Figure 9). In fact, according to Boumans et al. (2018) [42], growing pigs living in groups will have most of their fights during the competition for food resources, so it is more suitable to see this kind of contact in the area close to the feeder, rather than in the intermediate or resting areas. Accordingly, the feeding area was the only place of the pen where more negative than positive social interactions were seen (Figure 9). In fact, as described in other studies [43], positive social behavior was seen more frequently than negative social behavior.

Although statistical differences were found in the positive/negative social behavior ratio in relation to moment of the day, floor type, number of feeders, and even thermal conditions, numerically these differences were small (positive social behavior ranging from 54% to 59%). However, this was not the case for the place of the pen, as in the feeding area, positive social behavior was just 43% of the total, and in the resting area it was 65% of the total. Hyun and Ellis (2001) [44] observed a significantly higher number of displacements from the feeder in group sizes of 8 and 12 pigs (18.8% and 32.8% of observations, respectively) compared to group sizes of 2 and 4 pigs (5% of observations), and a higher competition for food (a single feeder was provided in all cases) was used as the explanation. Therefore, one of the objectives of the study, to reduce the competition for food by providing a double feeder, was achieved. Nevertheless, the effect on performance was contrary to what was expected. Instead of increasing weight gains, the double feeder resulted in a lower body weight, in comparison to pigs reared with just one feeder. One possible explanation is the effect of the reduction of available space in the pens when a supplementary feeder is provided, from 0.82 m^2^ to 0.79 m^2^ per pig. Although increased stocking densities have been described as risk factors in pigs due to more competition for space [45], this difference of 3.5% between treatments, by itself, probably is not enough to explain the results found. In fact, the differences appeared very early in the growing period, animals weighing just 50 kg, where 0.7 m^2^ is considered an optimal space even under heat conditions, when all pigs need to be lying fully recumbent [46]. Therefore, another explanation is needed.

Hyun and Ellis (2001, and 2002) and Nielsen et al. (1995) [44,47,48] described how meal duration and meal size increased when group size grew in a pen from 4 to 8 pigs and from 8 to 10–15 pigs. This is in agreement with the results found in the present study, where animals with two feeders had shorter meals than did those with just one. Thus, a possible explanation for the differences found could be that animals with one feeder may be forced to eat bigger meals due to more competition in the access to the feeder and, consequently, they had bigger stomachs and higher body weights than the animals with two feeders, with reduced meal sizes. However, this explanation is speculative. Unfortunately, with the video recordings done in the study, it was not possible to identify the animals individually, so it was not possible to establish an accurate frequency of meals per day for the different treatments. Therefore, the question of why competition was reduced with two feeders but weight gains did not improve remains unclear.

In relation to the hematological parameters, differences between treatments were found only for the white blood cells. However, in all cases except for lymphocytes, the differences found were inside the reference ranges for the species (Table 3). In the case of lymphocytes, a 100% slatted floor showed a higher value than expected in healthy animals [27]. In addition, neutrophils and total white blood cells were also higher in 100% slat than 30% slat 70% concrete, confirming again that this last type of floor was better for the pigs studied in the present study. According to the packed cell volume, higher than the reference values for pigs [27], all treatments suffered from some hemoconcentration (Table 3). This could be explained by heat stress [49], and although pigs had water ad libitum, they could have suffered from some dehydration.

According to St-Pierre et al. (2003) [50], the main problems of meat quality associated with heat stress is a decrease in lipid and protein content, in addition to processing carcass problems due to sub-optimal growth and inconsistent market weights. Chronic exposure of growing pigs to a high ambient temperature is associated with enhanced lipid metabolism in the liver and the adipose tissue. As a consequence, plasma triglyceride uptake and storage are facilitated in the adipose tissue, which results in greater fatness [51,52]. Increased fatness in long-term heat-exposed pigs is accompanied by changes in the distribution of adipose tissue: a shift of body fat toward internal sites [53]. The change in fat distribution in pigs exposed to high temperatures would appear as an adaptation to increase heat loss [50,51]. Accordingly, in the present study animals housed under control conditions had a higher lean percentage (less fat) than animals under heat stress conditions, with a mean difference of 2.6% between them.

Another factor influenced by environmental temperatures was ultimate pH. However, in both treatments, values were within the normal values for this parameter (from 5.4 to 5.7) and far from 6.0 to 6.4, which would lead the muscle to be DFD (Dark, Firm and Dry) pork meat [54,55]. Other parameters such as carcass yield, intramuscular fat, pH_1_, EC_1_, EC_u_, color, drip loss, and shear force were not affected by environmental temperature treatment, floor type, or number of feeders in the pen, so the results on meat quality were very limited in the present study.

## 5. Conclusions

Pigs subjected to temperatures above 30 °C up to 6 h a day had worse neutrophil/lymphocyte ratios, hair cortisol values, and body-weight gains than pigs subjected to a maximum temperature of 25 °C. This confirms that thermal stress negatively affects performance in pigs, especially during the last phase of the growing period, when animals are bigger and space is more limited. In addition, heat stress affects the final product by decreasing the lean percentage by 2.6%. A floor of 70% concrete and 30% slats results, as well, in higher body weights than does a 100% slat. Nonetheless, providing a double feeder in the pens, although it reduces the presence of negative social interactions, especially in the area around the feeder, negatively affects body weight, in comparison to pigs fed with just one feeder. In consequence, it is concluded that reducing competition for food does not improve animal production results in the conditions used in the present study.

## Figures and Tables

**Table 1:** Results of the statistical analysis (*p* = *p*-value) on the initial body weight (kg) and the average daily gain from this weight (W1) to one month later (W2), two months later (W3), and three months later (W4), and where floor is related to the type of floor (30% slat 70% concrete vs. 100% slat), heat to the effect of temperature (heat room vs. control room), and feeder to the number of feeders per pen (one: single; two: double), and all the possible double (floorxheat, floorxfeeder, and heatxfeeder) and triple (floorxheatxfeeder) interactions.

Variable	Floor			Heat			Feeder			Floor × Heat	Floor × Feeder	Heat × Feeder	Floor × Heat × Feeder
	30% Slat	100% Slat	*p*	Control	Heat	*p*	One	Two	*p*	*p*	*p*	*p*	*p*
Initial body weight (kg)	27.5	27.7	0.6885	27.5	27.8	0.5265	27.9	27.4	0.1857	0.8911	0.8720	0.7915	0.9990
Average daily gain (kg) from W1 to W2	0.79	0.76	0.1345	0.79	0.76	0.1877	0.80	0.75	0.0377	0.6301	0.5921	0.5313	0.6243
Average daily gain (kg) from W2 to W3	0.82	0.82	0.3246	0.82	0.83	0.2391	0.84	0.80	0.0135	0.7872	0.7730	0.7115	0.6384
Average daily gain (kg) from W3 to W4	1.01	0.95	<0.001	1.01	0.96	0.0245	1.00	0.96	0.0443	0.9536	0.5276	0.7315	<0.001

The significance is fixed in all cases at *p* < 0.05.

**Table 2:** Results of the statistical analysis of the different variables considered in the study, where heat is related to the effect of temperature (heated room vs. control room), floor to the type of floor (30% slat 70% concrete vs. 100% slat), and feeder to the number of feeders per pen (one: single; two: double), and all the possible double (heatxfloor, heatxfeeder and floorxfeeder) and triple (heatxfloorxfeeder) interactions.

Variable	Heat	Floor	Feeder	Heat × Floor	Heat × Feeder	Floor × Feeder	Heat × Floor × Feeder
Final body weight	<0.0001	0.0387	0.0234	NS	NS	NS	<0.0001
Packed cell volume	NS	NS	NS	NS	NS	NS	NS
Hemoglobin	NS	NS	NS	NS	NS	NS	NS
Total red blood cells	NS	NS	NS	NS	NS	NS	NS
Total white blood cells	NS	0.0037	NS	NS	NS	NS	NS
Neutrophils	NS	0.0023	NS	NS	NS	NS	NS
Lymphocytes	NS	0.0008	NS	NS	NS	NS	NS
Eosinophils	0.0012	NS	NS	NS	NS	NS	NS
Basophils	NS	NS	NS	NS	NS	NS	NS
Monocytes	0.0006	NS	NS	NS	NS	NS	NS
NLR	NS	NS	NS	0.0007	NS	NS	NS
Hair cortisol	NS	NS	NS	0.0492	NS	NS	NS
Positive social behavior	0.0013	NS	NS	0.0030	NS	NS	NS
Negative social behavior	NS	0.0005	0.0008	NS	NS	NS	NS
Carcass yield	NS	NS	NS	NS	NS	NS	NS
Lean meat percentage	0.0434	NS	NS	<0.0001	NS	NS	NS
Intramuscular fat	NS	NS	NS	NS	NS	NS	NS
pH_1_	NS	NS	NS	NS	NS	NS	NS
pH_u_	0.0094	NS	NS	NS	NS	NS	NS
EC_1_	NS	NS	NS	NS	NS	NS	NS
EC_u_	NS	NS	NS	NS	NS	NS	NS
L*	NS	NS	NS	NS	NS	NS	NS
Drip Loss	NS	NS	NS	NS	NS	NS	NS
Shear force	NS	NS	NS	NS	NS	NS	NS

NLR—neutrophil/lymphocyte ratio; pH_1_—pH after one hour post-mortem; pH_u_—pH after 24 h post-mortem; EC_1_—electrical conductivity at one hour post-mortem; EC_u_—electrical conductivity after 24 h post-mortem; L*—meat lightness determined at 24 h post-mortem. The significance is fixed in all cases at *p* < 0.05. NS = Not significant differences.

**Table 3:** Effect of temperature (heat room vs. control room), type of floor (30% slat 70% concrete vs. 100% slat), and number of feeders (one: single; two: double) on hematological parameters of pigs at the end of the study.

	Reference Values *	Temperature		Floor		Feeder	
Variable		Heated	Control	30%	100%	Single	Double
Packed cell volume (%)	36–47	50.2 ± 0.29	50.6 ± 0.31	50.4 ± 0.29	50.4 ± 0.28	50.3 ± 0.29	50.5 ± 0.27
Hemoglobin (g/dL)	10–15	12.0 ± 0.08	12.1 ± 0.09	12.1 ± 0.07	12.1 ± 0.08	12.0 ± 0.07	12.1 ± 0.08
Red blood cells (10^6^/μL)	5–10	7.3 ± 0.35	7.3 ± 0.39	7.3 ± 0.37	7.3 ± 0.37	7.3 ± 0.35	7.4 ± 0.36
White blood cells (10^3^/μL)	6–25	22.8 ± 0.44	23.6 ± 0.44	22.0 ± 0.46 ^b^	24.5 ± 0.46 ^a^	23.4 ± 044	23.2 ± 0.49
Neutrophils (10^3^/μL)	2–8.85	8.18 ± 0.215	8.02 ± 0.221	7.74 ± 0.207 ^b^	8.23 ± 0.219 ^a^	8.18 ± 0.213	8.08 ± 0.219
Lymphocytes (10^3^/μL)	4–13.8	12.8 ± 0.567	13.5 ± 0.561	12.4 ± 0.557 ^b^	14.3 ± 0.573 ^a^	13.2 ± 0.572	13.2 ± 0.569
Eosinophils (10^3^/μL)	0.18–1.32	0.49 ± 0.389 ^b^	0.69 ± 0.433 ^a^	0.60 ± 0.413	0.58 ± 0.379	0.58 ± 0.398	0.59 ± 0.376
Basophils (10^3^/μL)	0–0.47	0.21 ± 0.170	0.19 ± 0.177	0.21 ± 0.175	0.20 ± 0.173	0.22 ± 0.176	0.21 ± 0.179
Monocytes (10^3^/μL)	0.3–2.03	1.19 ± 0.428 ^a^	1.02 ± 0.425 ^b^	1.08 ± 0.424	1.18 ± 0.431	1.20 ± 0.428	1.14 ± 0.427

* Reference values according to Mitruka and Rawsnley, (1977) [27]. Different letters in the same treatment row mean significance at *p* < 0.01.

**Table 4:** Effect of temperature (heated room vs. control room), type of floor (30% slat 70% concrete vs. 100% slat), and number of feeders (one: single; two: double) on carcass and meat-quality parameters.

	Temperature		Floor		Feeder	
Variable	Heat Stress	Control	30%	100%	Single	Double
Carcass yield (%)	75.1 ± 0.65	74.8 ± 1.02	75.6 ± 0.44	74.2 ± 1.24	75.1 ± 0.87	74.8 ± 0.81
Lean meat percentage	49.6 ± 0.86 ^b^	52.2 ± 1.35 ^a^	50.2 ± 0.58	51.6 ± 1.65	50.3 ± 1.13	51.5 ± 1.07
Intramuscular fat (%)	4.0 ± 0.11	3.9 ± 0.09	3.9 ± 0.09	4.0 ± 0.10	4.0 ± 0.11	4.0 ± 0.14
pH_1_	6.5 ± 0.14	6.5 ± 0.22	6.5 ± 0.11	6.5 ± 0.26	6.5 ± 0.20	6.4 ± 0.18
pH_u_	5.5 ± 0.04 ^b^	5.7 ± 0.03 ^a^	5.6 ± 0.03	5.6 ± 0.04	5.7 ± 0.10	5.5 ± 0.09
EC_1_ (mS)	3.5 ± 0.19	3.6 ± 0.16	3.8 ± 0.16	3.3 ± 0.18	3.5 ± 0.20	3.6 ± 0.24
EC_u_ (mS)	3.0 ± 0.13	3.2 ± 0.11	3.0 ± 0.11	3.1 ± 0.12	3.0 ± 0.13	3.2 ± 0.16
L*	46.4 ± 0.81	45.7 ± 0.72	46.3 ± 0.71	45.8 ± 0.78	45.6 ± 0.86	46.5 ± 1.06
Drip Loss (%)	0.9 ± 0.37	1.1 ± 0.31	1.0 ± 0.34	1.0 ± 0.37	0.7 ± 0.41	1.3 ± 0.50
Shear force (kg)	5.9 ± 0.62	5.2 ± 0.54	5.5 ± 0.47	5.6 ± 0.56	5.6 ± 0.61	5.6 ± 0.78

Data presented in mean ± SE. pH_1_—pH after 1 h post-mortem, pH_u_—pH after 24 h post-mortem. EC_1_—electrical conductivity at 1 h post-mortem, EC_u_—electrical conductivity after 24 h post-mortem, L*—meat lightness determined at 24 h post-mortem. Different letters in the same treatment row mean significance at *p* < 0.05.
